# Impact of *CYP1A1* Polymorphisms on Susceptibility to Chronic Obstructive Pulmonary Disease: A Meta-Analysis

**DOI:** 10.1155/2015/942958

**Published:** 2015-09-03

**Authors:** Cheng-Di Wang, Nan Chen, Lin Huang, Jia-Rong Wang, Zhi-Yuan Chen, Ya-Mei Jiang, Ya-Zhou He, Yu-Lin Ji

**Affiliations:** ^1^Department of Respiratory Medicine, West China Hospital, Sichuan University, Chengdu, Sichuan 610041, China; ^2^West China School of Medicine/West China Hospital, Sichuan University, Chengdu, Sichuan 610041, China

## Abstract

*Objective. *Several studies have evaluated the association between* CYP1A1 *polymorphisms and the susceptibility of chronic obstructive pulmonary disease (COPD) with inconclusive results. We performed the first comprehensive meta-analysis to summarize the association between* CYP1A1 *polymorphisms and COPD risk.* Method. *A systematic literature search was conducted (up to April 2015) in five online databases: PubMed, EMBASE, China National Knowledge Infrastructure (CNKI), WeiPu, and WanFang databases. The strength of association was calculated by odds ratio (OR) and corresponding 95% confidence interval (CI).* Results. *Seven case-control studies with 1050 cases and 1202 controls were included. Our study suggested a significant association between the MspI polymorphism and COPD risk (CC versus TC + TT: OR = 1.57, CI: 1.09–2.26, *P* = 0.02; CC versus TT: OR = 1.73, CI: 1.18–2.55, *P* = 0.005). For the Ile/Val polymorphism, a significant association with COPD risk was observed (GG versus AG + AA: OR = 2.75, CI: 1.29–5.84, *P* = 0.009; GG versus AA: OR = 3.23, CI: 1.50–6.93, *P* = 0.003; AG versus AA: OR = 1.39, CI: 1.01–1.90, *P* = 0.04). Subgroup analysis indicated a significant association between the MspI variation and COPD risk among Asians (CC versus TC + TT: OR = 1.70, CI: 1.06–2.71, *P* = 0.03; CC versus TT: OR = 1.84, CI: 1.11–3.06, *P* = 0.02).* Conclusion. *The MspI and Ile/Val polymorphisms might alter the susceptibility of COPD, and MspI polymorphism might play a role in COPD risk among Asian population.

## 1. Introduction

Chronic obstructive pulmonary disease (COPD) represents the fourth leading cause of death worldwide. There are over 40 million patients with COPD in China and over 1.28 million patients die of COPD per year [1]. The pathogenesis of COPD was affected by the multifactorial interactions of both environmental triggers and genetic susceptibility [2, 3]. In addition to other contributing factors including airway hyperresponsiveness, family history of asthma, chronic inflammation, and childhood respiratory infections [4, 5], environmental exposure like tobacco smoke absorption, which plays role in increasing goblet cells count, airway remodeling, and tissue destructing, is the most commonly recognized risk factor for COPD. However, Fletcher and Peto [6] suggested that only 10% to 15% smokers would develop to symptomatic COPD, revealing the susceptibility to tobacco smoke injury may be related to genetic variety among individuals. Up to now, a wide variety of researches had presented that single-nucleotide polymorphism (SNP) might have an effect on the quantity or activity of a specific protein, thus making individual and hereditary contribution to the COPD development [7–9].

Polycyclic aromatic hydrocarbons (PAHs), carcinogen mainly from cigarette smoking and air pollution, have been proved to be related to the development of COPD [10, 11]. One of PAH-metabolizing cytochrome P450 enzymes, CYP1A1, is a well-known aryl hydrocarbon hydroxylase, widely expressed in tissues including liver, lung, intestine, skin, lymphocytes, and macrophages [12]; many studies had investigated the carcinogenic role of these CYPs in related pulmonary disease including COPD [13–17]. Located in chromosome 15q22–24,* CYP1A1* gene contained lots of SNPs, among which MspI and Ile/Val polymorphisms were most widely studied.* CYP1A1* MspI polymorphism, which resulted in a T-C change in the 30-non-coding region of* CYP1A1*, might link to some other functional polymorphisms and thus has been reported to be associated with an increased enzyme activity [18–20]. Similarly, adenine to guanine transition at position 2455 in* CYP1A1* exon 7 also resulted in the substitution of Val for Ile in the enzyme active center [21]. Hence, these SNPs in* CYP1A1* gene might lead to an altered enzyme activity of detoxicating cigarette smoke products and thus be related to lung function and susceptibility of COPD [22, 23]. Moreover, lots of studies have reported that polymorphisms of* CYP1A1* gene could modify susceptibility to smoking-induced lung cancer [15, 24].

A few studies [25–31] have investigated the association between* CYP1A1* gene polymorphisms and COPD risk. However, their results were inconclusive partly because of limitation of population size, ethnicities, and other confounding factors. Thus we performed the first comprehensive meta-analysis including all eligible case-control studies to date, to assess the exact effect of* CYP1A1* MspI and Ile/Val polymorphisms on COPD risk.

## 2. Materials and Methods

### 2.1. Search Strategy

We performed a comprehensive search up to April 13, 2015, without language limitation in the following five electronic databases: PubMed, EMBASE, China National Knowledge Infrastructure (CNKI), Weipu, and WanFang databases. The following words were used as search terms: “COPD” or “chronic obstructive pulmonary disease” and “CYP1A1” or “cytochrome P4501A1” or “P4501A1” and “polymorphism” or “variant” or “mutation.” Reference lists of relevant articles were also checked manually for potential eligible studies.

### 2.2. Inclusion and Exclusion Criteria

Studies were included if they met the following criteria: (1) case-control studies; (2) investigating the association between* CYP1A1* gene polymorphisms and susceptibility to COPD; (3) detailed genotype frequencies that were available to calculate odds ratios (OR) with a 95% confidence interval (CI). Studies were excluded if they were (1) conference abstracts, animal studies, cellular studies, or letters; (2) without available genotype frequencies; (3) duplication publications. When studies with overlapped case series were met, we included the studies with maximal subjects.

### 2.3. Data Extraction

With a standard extraction table, two investigators (Lin Huang and Nan Chen) extracted the data independently. The following information was collected from each eligible study: first author' name, year of publication, country, ethnicity of subjects, source of controls (hospital-based or population-based), matching criteria for controls, genotyping method, and genotype frequency distribution of cases and controls. These two investigators launched a final discussion to reach a consensus if any disagreement was met.

### 2.4. Statistical Analysis

Person's Chi-square was conducted to assess the Hardy-Weinberg equilibrium of genotype frequencies in controls of each eligible study. *P* value less than 0.05 was regarded as significant disequilibrium. The data was excluded from the susceptibility analysis if significant disequilibrium existed. The strength of association between* CYP1A1* polymorphisms and susceptibility to COPD was evaluated by calculating the odds ratio (OR) with corresponding 95% confidence interval (CI) under the following five genetic models: dominant model, recessive model, homozygote comparison, heterozygote comparison, and allele model. To assess the significance of OR, we conducted the *Z* test and we regarded it as significant difference when *P* value less than 0.05 was detected. Moreover, *χ* ^2^ based *Q* and *I* ^2^ test were performed to evaluate the between-study heterogeneity and *P* < 0.1 was defined as statistical significance. The random effect was used to calculate the OR if significant heterogeneity existed. Otherwise, the fixed-effects model was applied. Furthermore, we also conducted subgroup analyses stratified by ethnicity (Caucasian and Asian), source of controls (population-based and hospital-based), matching criteria (more than 2 items and less than 3 items), and sample size (larger than 300 and less than 300). Publication bias was assessed by asymmetry of funnel plots. In sensitivity analysis, we sequentially excluded each study on the software to evaluate the stability of the results. We conducted all the analyses by using software Review Manager 5.2.

## 3. Results

### 3.1. Baseline Characteristics of Included Studies

**Figure 1:**
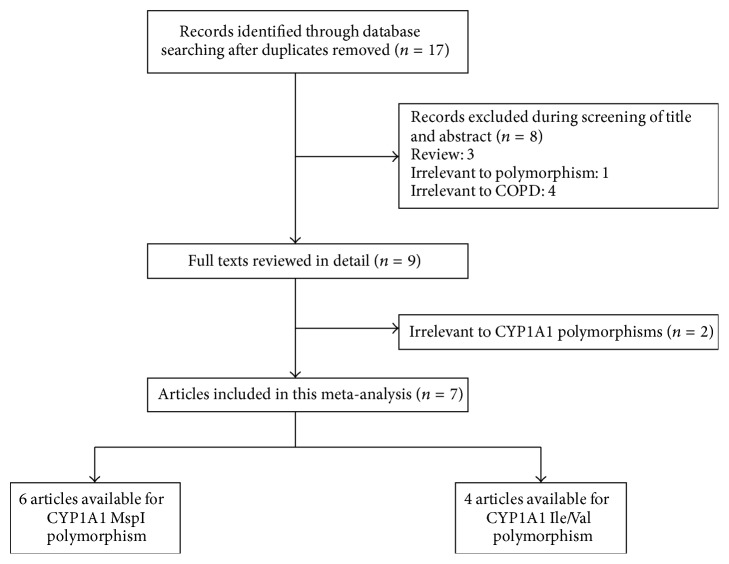
The flowchart of literature search and study selection.

Seventeen records were identified after searching these five databases mentioned above (Figure 1). After reviewing of title and abstract, 9 records were considered as potentially eligible and full texts were retrieved. After screening the full texts, we included seven studies which met our inclusion and exclusion criteria. Finally, a total of seven studies with 1050 cases and 1202 controls were included in our meta-analysis. When investigating association between Ile/Val polymorphism and COPD risk, Gaspar et al. [25] recruited controls apart from Hardy-Weinberg equilibrium and these data were excluded from susceptibility analysis. Thus there were six studies for MspI polymorphism and four studies for Ile/Val polymorphism. As for ethnicity, two studies were carried out among Caucasians and five studies were performed among Asians. Of eligible studies, three studies recruited controls from population and three studies recruited hospital-based controls. The detailed characteristics of eligible studies were presented in Table 1 and the genotype frequency distribution was shown in Table 2.

### 3.2. Meta-Analysis Results

**Figure 2:**
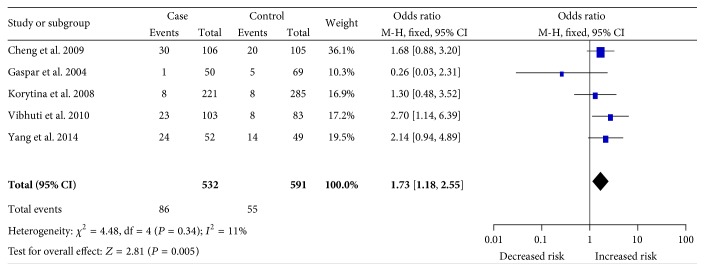
Forest plot for the association between the* CAP1A1* MspI polymorphism and susceptibility to COPD under the homozygote comparison (CC versus TT). Significant association was observed between the MspI polymorphism and increased COPD risk.

**Figure 3:**
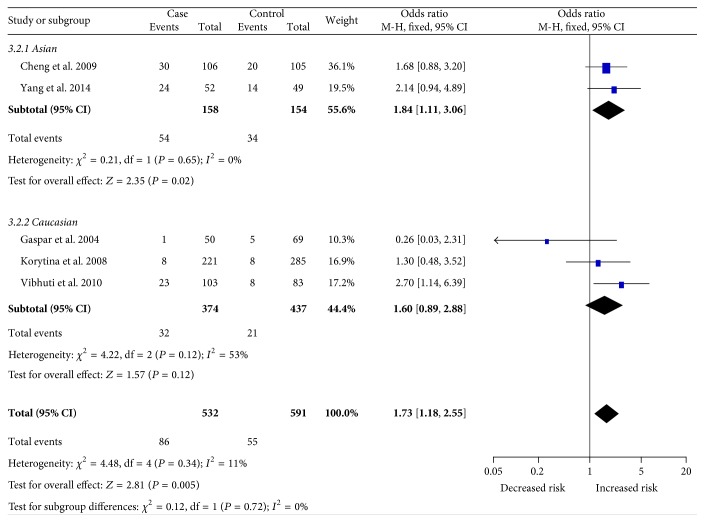
Forest plot of subgroup analysis for the association between the* CAP1A1* MpsI polymorphism and COPD risk among Asians (CC versus TT). We observed significant association between the MpsI polymorphism and COPD risk among Asians.

Overall, we observed a significant association between MspI polymorphisms and increased COPD risk (CC versus TC + TT: OR = 1.57, CI: 1.09–2.26, *P* = 0.02; CC versus TT: OR = 1.73, CI: 1.18–2.55, *P* = 0.005; Figure 2). In subgroup analysis stratified by ethnicity, a significant association between MspI polymorphisms and susceptibility to COPD was detected among Asians (CC versus TC + TT: OR = 1.70, CI: 1.06–2.71, *P* = 0.03; CC versus TT: OR = 1.84, CI: 1.11–3.06, *P* = 0.02; Figure 3). Furthermore, we also found a significant association in meta-analysis among studies with hospital-based controls (TC + CC versus TT: OR = 2.01, CI: 1.40–2.87, *P* < 0.001), matching criteria (more than 2 items) (CC versus TT: OR = 2.01, CI: 1.06–3.81, *P* = 0.03), and sample size (more than 300) (CC versus TT: OR = 1.84, CI: 1.17–2.89, *P* = 0.009). The detailed results for subgroup analysis were shown in Table 3.

**Figure 4:**
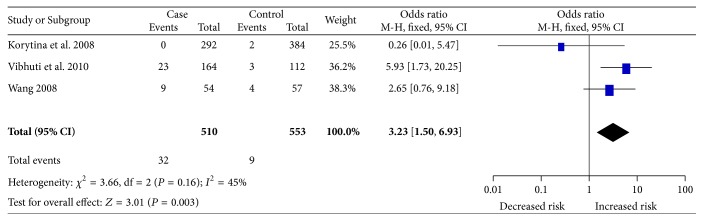
Forest plot for the association between the* CAP1A1* Ile/Val polymorphism and susceptibility to COPD under the homozygote comparison (GG versus AA). Significant association was observed between the Ile/Val polymorphism and increased COPD risk.

Totally, a significant association between Ile/Val polymorphism and COPD risk was identified (GG versus AG + AA: OR = 2.75, CI: 1.29–5.84, *P* = 0.009; GG versus AA: OR = 3.23, CI: 1.50–6.93, *P* = 0.003; AG versus AA: OR = 1.39, CI: 1.01–1.90, *P* = 0.04; Figure 4). As for ethnicity, we did not detect any significant association between Ile/Val polymorphism and susceptibility to COPD among both Asian and Caucasian population. When stratified by source of controls, we observed a significant relationship with Ile/Val polymorphism and COPD risk among studies recruiting hospital-based controls (AG + GG versus AA: OR = 1.99, CI: 1.37–2.90, *P* < 0.001; Table 3).

### 3.3. Publication Bias and Sensitivity Analysis

**Figure 5:**
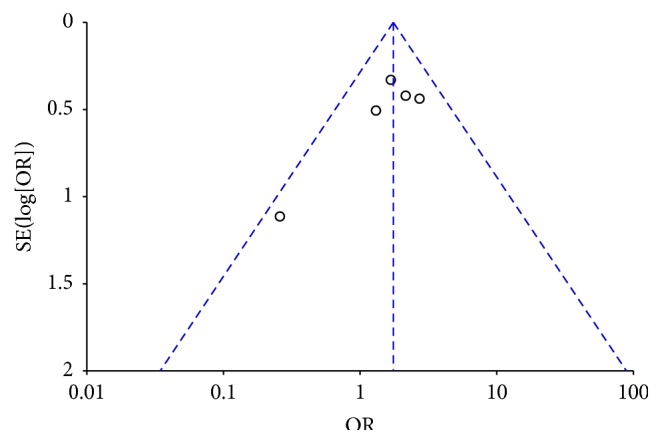
Begg's funnel plot on publication bias for included studies on the association of the* CAP1A1* MspI and Ile/Val polymorphisms with susceptibility to COPD. The symmetrical shape of the funnel plot indicated absence of publication bias.

Publication bias was evaluated by funnel plots. The shape of the funnel plots seemed symmetric and it indicated no significant publication bias in our meta-analysis (Figure 5). Sensitivity analysis was also conducted by excluding individual studies sequentially. The results of meta-analysis did not change qualitatively by any single study, suggesting the stability of our results.

## 4. Discussion

CYP1A1 is a well-known aryl hydrocarbon hydroxylase in CYP1 family; its function in xenobiotic biotransformation and antioxidant defense are crucial in protecting lungs from toxic agents [32, 33]. The variations in structure and function of CYP1A1 on account of MspI and Ile/Val polymorphisms might result in altered CYP1A1 enzyme activity and different rate of procarcinogens metabolism like PAHs [10], nitrosamines, and aromatic amines [19], thus generating individual's risk difference when exposed to environmental COPD inducers. Therefore, it is reasonable to speculate that impairment of lung function and pathogenesis of COPD may develop in individuals carrying the variant MspI and Ile/Val in CYP1A1. To eliminate existing shortages in published researches such as small simple size and other cofounding factors and to evaluate the exact relationship with CYP1A1 polymorphisms and COPD risk, we conducted the present meta-analysis of seven eligible case-control studies. Our result showed a significant association between both MspI and Ile/Val polymorphism and susceptibility to COPD, and stratified analyses showed that MspI polymorphism might also be related to increased COPD risk among Asians.

As one of our conclusions, significant association between MspI polymorphism and susceptibility to COPD was detected. Individuals with CC genotype of MspI polymorphism seemed to bear higher risk of COPD than that of TT genotype. In subgroup analysis, these results were also observed among Asians and a 1.84-fold higher risk was detected in individuals with CC genotype compared to TT genotype. The significant relationship was also identified in meta-analysis among studies with more matching criteria and larger sample size (Table 3). These results indicated that the relationship between MspI polymorphism and risk of COPD in terms of Caucasian was further explored, and more well-designed studies with larger subjects were warranted. For Ile/Val polymorphism, a significant association between this polymorphism and COPD risk was also detected. Both AG and GG genotype might mean higher COPD risk than AA genotype. However, we did not find any association among both Asian and Caucasian population, which might be a result of relatively smaller sample size in subgroup analysis, which reflected the effective role of our meta-analysis and more studies were hoped in future; thus we could further examine the exact effect among ethnicities. Moreover, functional studies on the exact molecular mechanism were hoped to conduct in future. Although these results might be limited by quantity of included studies, we still believed a significant trend existed between* CYP1A1* polymorphisms and COPD risk and our meta-analysis figured out the direction of further studies. MspI and Ile/Val polymorphisms might be focused for more investigation as important genetic biomarkers, which might play a great role in development, diagnosis, treatment, and prognosis of COPD.

Moreover, effects of* CYP1A1* polymorphisms on COPD risk among both smokers and nonsmokers were worth evaluating. However, only few studies [30] assessed this issue and the data was insufficient to conduct meta-analysis. We expected more studies involved in this issue in future. Another important thing was that Cheng et al. [27] reported a significant association between MspI polymorphism and severity of COPD, and variant allele was higher among severe COPD patients. However, Putra et al. [29] indicated no relationship with* CYP1A1* polymorphisms and severity of COPD in their study. These data pieces could not be synthesized for different criteria of COPD severity used in their studies. Considering the results of our study, we hypothesized that variant allele of MspI polymorphism might contribute to more severe chronic inflammation and oxidative stress of lung and thus might be related to not only susceptibility to COPD but also further progression of COPD. Putra et al. [29] did not detect any association and it might be partly because of limitation of relatively smaller sample size. More studies were warranted to focus on this hypothesis and identify the finite conclusion.

There were some limitations that should be presented. First, only a few studies were published and included yet. More studies investigating these issues were warranted in future. Second, effect of Ile/Val polymorphism on COPD risk among other ethnicities was unavailable for lack of sufficient data. Further studies on Ile/Val polymorphism on COPD risk among different races were hoped. Third, our records were identified in specific standard databases in English and Chinese, some potential eligible studies in other languages might be left, and even though the possibility of omission was minimized, the publication bias might influence the results of this meta-analysis that cannot be absolutely excluded.

## 5. Conclusion

Our meta-analysis demonstrated a significant association between both MspI and Ile/Val polymorphisms of* CYP1A1* gene and susceptibility to COPD. MspI polymorphism might also be related to increased COPD risk among Asians. Overall, in terms of the pathogenesis of COPD, genetic polymorphisms should be simultaneously considered to accomplish the comprehensive mechanism of varied environmental inducer sensitivity, which may be valued in individualized prevention and varied prognosis of COPD associated with environmental exposures. More well-designed studies with larger sample size were warranted.

## Figures and Tables

**Table 1:** Baseline characteristics of eligible case-control studies.

First author	Year	Reference	Country	Ethnicity	Source of controls	Matching criteria for controls	Cases/controls	Quality control	Polymorphisms
Cheng	2009	[27]	China	Asian	PB	Age	184/212	NA	MspI,
Gaspar	2004	[25]	Brazil	Caucasian	PB	NA	75/90	NA	MspI, Ile/Val
Korytina	2008	[26]	Russia	Caucasian	PB	Age, sex, ethnicity	319/418	NA	MspI, Ile/Val
Putra	2013	[29]	Japan	Asian	HB/PB	Sex, ethnicity	48/172	Yes	MspI, Ile/Val
Vibhuti	2010	[28]	India	Caucasian	HB	Sex, ethnicity, area	210/136	NA	MspI, Ile/Val
Wang	2008	[31]	China	Asian	HB	Age, sex	115/94	NA	Ile/Val
Yang	2014	[30]	China	Asian	HB	Age, sex	101/80	NA	MspI

PB: population-based; HB: hospital-based; HB/PB: recruiting both population-based and hospital-based controls; NA: not available.

**Table 2:** Genotype frequency distribution of *CYP1A1* polymorphisms.

Polymorphisms	First author	Case				Control				HWE in controls	MAF in controls
		*N* ^a^	TT	TC	CC	*N* ^a^	TT	TC	CC		

MspI (rs4646903)	Cheng	184	76	78	30	212	85	107	20	2.76	0.35
	Gaspar	75	49	25	1	90	64	21	5	2.97	0.17
	Korytina	275	213	54	8	387	277	102	8	0.15	0.15
	Putra	48	21	27		172	57	115		NA	NA
	Vibhuti	210	80	107	23	136	75	53	8	0.12	0.25
	Yang	101	28	49	24	80	35	31	14	2.24	0.37

		*N* ^a^	AA	AG	GG	*N* ^a^	AA	AG	GG		

Ile/Val (rs1048943)	Gaspar	75	59	16	0	90	73	14	3	4.06^b^	0.11
	Korytina	317	292	25	0	418	382	34	2	1.64	0.05
	Putra	48	28	20		172	93	79		NA	NA
	Vibhuti	210	141	46	23	136	109	24	3	1.38	0.11
	Wang	115	45	61	9	94	53	37	4	0.62	0.24

^a^Sample size of case group or control group.

^b^Gaspar recruited controls apart from Hardy-Weinberg equilibrium and thus these data were excluded from susceptibility analysis.

*N*: sample size in case or control group; NA: not available; HWE: Hardy-Weinberg equilibrium; MAF: minor allele frequency.

**Table 3:** The results of evidence synthesis in this meta-analysis.

Variables	Dominant model				Recessive model				Homozygote comparison				Heterozygote comparison				Additive model			
	*N* ^a^	OR (CI)	*P*	*P*_*h*_	*N*	OR (CI)	*P*	*P*_*h*_	*N*	OR (CI)	*P*	*P*_*h*_	*N*	OR (CI)	*P*	*P*_*h*_	*N*	OR (CI)	*P*	*P*_*h*_
*MspI*																				
Total	6	1.14 (0.77, 1.70)	0.51	0.002	5	1.57 (1.09, 2.26)	0.02	0.45	5	1.73 (1.18, 2.55)	0.005	0.34	5	1.22 (0.77, 1.94)	0.4	0.002	5	1.21 (0.91, 1.60)	0.2	0.02
By ethnicity																				
Asian	3	1.07 (0.59, 1.92)	0.83	0.03	2	1.70 (1.06, 2.71)	0.03	0.62	2	1.84 (1.11, 3.06)	0.02	0.65	2	1.22 (0.51, 2.89)	0.65	0.03	2	1.29 (0.94, 1.78)	0.06	0.2
Caucasian	3	1.23 (0.63, 2.40)	0.55	0.002	3	1.39 (0.78, 2.48)	0.27	0.19	3	1.60 (0.89, 2.88)	0.12	0.12	3	1.24 (0.61, 2.51)	0.55	0.002	3	1.13 (0.69, 1.86)	0.62	0.008
By source of controls																				
PB	3	0.88 (0.69, 1.12)	0.3	0.28	3	1.48 (0.91, 2.41)	0.11	0.18	3	1.35 (0.81, 2.24)	0.25	0.27	3	0.82 (0.64, 1.07)	0.14	0.13	3	0.98 (0.80, 1.20)	0.85	0.31
HB	2	2.01 (1.40, 2.87)	0.0001	0.97	2	1.68 (0.97, 2.91)	0.06	0.61	2	2.40 (1.32, 4.36)	0.004	0.71	2	1.92 (1.32, 2.80)	0	0.92	2	1.65 (1.26, 2.14)	0.0002	0.82
By matching criteria																				
More than 2 items	2	1.09 (0.83, 1.44)	0.52	0.0005	2	1.73 (0.92, 3.27)	0.09	0.62	2	2.01 (1.06, 3.81)	0.03	0.28	2	1.13 (0.42, 3.05)	0.81	0.0008	2	1.17 (0.57, 2.39)	0.67	0.002
Less than 3 items	4	1.09 (0.83, 1.43)	0.55	0.07	3	1.49 (0.95, 2.33)	0.08	0.18	3	1.59 (0.98, 2.58)	0.06	0.21	3	1.29 (0.73, 2.30)	0.39	0.06	3	1.23 (0.98, 1.53)	0.07	0.38
By sample size																				
>300	3	1.11 (0.62, 1.96)	0.73	0.002	3	1.80 (1.16, 2.79)	0.008	0.87	3	1.84 (1.17, 2.89)	0.009	0.52	3	1.01 (0.56, 1.83)	0.97	0.002	3	1.15 (0.77, 1.72)	0.49	0.008
<300	3	1.20 (0.62, 2.33)	0.6	0.04	2	1.13 (0.58, 2.20)	0.72	0.11	2	0.96 (0.13, 7.25)	0.97	0.07	2	1.76 (1.09, 2.84)	0.02	0.63	2	1.37 (0.98, 1.92)	0.07	0.26

*Ile/Val*																				
Total	4	1.34 (0.84, 2.13)	0.21	0.04	3	2.75 (1.29, 5.84)	0.009	0.15	3	3.23 (1.50, 6.93)	0.003	0.16	3	1.39 (1.01, 1.90)	0.04	0.21	3	1.50 (0.88, 2.55)	0.13	0.02
By ethnicity																				
Asian	2	1.32 (0.56, 3.11)	0.52	0.04																
Caucasian	2	1.34 (0.63, 2.88)	0.45	0.04	2	1.67 (0.09, 30.45)	0.73	0.07	2	1.72 (0.09, 34.41)	0.72	0.06	2	1.19 (0.81, 1.74)	0.37	0.27	2	1.41 (0.55, 3.63)	0.48	0.005
By source of controls																				
HB	2	1.99 (1.37, 2.90)	0.0003	0.96	2	3.48 (1.50, 8.08)	0.004	0.23	2	4.24 (1.79, 10.04)	0.001	0.36	2	1.69 (1.13, 2.51)	0.01	0.5	2	1.94 (1.43, 2.64)	0.0001	0.33
By matching criteria																				
More than 2 items	2	1.34 (0.63, 2.88)	0.45	0.04	2	1.67 (0.09, 30.45)	0.73	0.07	2	1.72 (0.09, 34.41)	0.72	0.06	2	1.19 (0.81, 1.74)	0.37	0.27	2	1.41 (0.55, 3.63)	0.48	0.005
Less than 3 items	2	1.32 (0.56, 3.11)	0.52	0.04																
By sample size																				
>300	2	1.34 (0.63, 2.88)	0.45	0.04	2	1.67 (0.09, 30.45)	0.73	0.07	2	1.72 (0.09, 34.41)	0.72	0.06	2	1.19 (0.81, 1.74)	0.37	0.27	2	1.41 (0.55, 3.63)	0.48	0.005
<300	2	1.32 (0.56, 3.11)	0.52	0.04																

^a^Number of included studies in evidence synthesis.

*P*: *P* value of *Z* test to evaluate the significance of the ORs; *P*_*h*_: *P* value of *Q* test for heterogeneity test.
